# Clinical impact of D3 lymph node dissection with left colic artery (LCA) preservation compared to D3 without LCA preservation: Exploratory subgroup analysis of data from JCOG0404

**DOI:** 10.1002/ags3.12318

**Published:** 2020-02-26

**Authors:** Tomonori Akagi, Masafumi Inomata, Takao Hara, Junki Mizusawa, Hiroshi Katayama, Dai Shida, Masayuki Ohue, Masaaki Ito, Yusuke Kinugasa, Yoshihisa Saida, Tadahiko Masaki, Seiichiro Yamamoto, Tsunekazu Hanai, Shigeki Yamaguchi, Masahiko Watanabe, Kenichi Sugihara, Haruhiko Fukuda, Yukihide Kanemitsu, Seigo Kitano

**Affiliations:** ^1^ Gastroenterological and Pediatric Surgery Oita University of Faculty of Medicine Yufu Japan; ^2^ Japan Clinical Oncology Group Data Center/Operations Office National Cancer Center Hospital Tokyo Japan; ^3^ Department of Colorectal Surgery National Cancer Center Tokyo Japan; ^4^ Department of Surgery Osaka International Cancer Institute Osaka Japan; ^5^ Department of Colorectal Surgery National Cancer Center Hospital East Kashiwa Japan; ^6^ Department of Gastrointestinal Surgery Tokyo Medical and Dental University Tokyo Japan; ^7^ Department of Colorectal Surgery Toho University Tokyo Japan; ^8^ Department of Surgery Kyorin University Tokyo Japan; ^9^ Department of Surgery Fujita Health University Aichi Japan; ^10^ Department of Gastroenterological Surgery Saitama Medical University International Medical Center Saitama Japan; ^11^ Department of Surgery Kitasato University School of Medicine Sagamihara Japan; ^12^ Department of Surgery Tokyo Medical and Dental University Tokyo Japan

**Keywords:** colon cancer, D3, left colic artery preserving, long‐term outcomes, postoperative complications

## Abstract

**Aim:**

We investigated the clinical impact of D3 lymph node dissection preserving left colic artery (LCA) compared to D3 without LCA preservation using data from JCOG0404. LCA preservation is expected to maintain adequate blood supply, which is effective in preventing anastomotic leakage, intestinal paralysis, and bowel obstruction. Whether D3 with LCA preservation (Group A) improves clinical outcomes following resection of sigmoid colon cancer compared to D3 without LCA preservation (Group B) is unclear.

**Methods:**

Procedure type was identified from photographs of the surgical field collected for central surgical review in JCOG0404. Clinical outcomes were compared between each procedure.

**Results:**

Among the 1057 randomized patients in JCOG0404, 631 patients receiving sigmoid colectomy or anterior resection were included in the subgroup analysis. Group A comprised of 135 patients and Group B of 496 patients. Patient backgrounds did not differ between groups. Median operative time, blood loss, anastomotic leakage, and intestinal paralysis were not remarkably different (Group A vs Group B: 185 vs 186 minutes, 60 vs 50 mL, 3.0% vs 5.0%, and 2.2% vs 3.8%). More overall postoperative complications occurred in Group B than Group A (21.6% vs 9.6%, *P* = .022). Five‐year relapse‐free survival (RFS) and overall survival (OS) tended to be better in Group A than Group B (RFS: 83.7% and 80.5%, HR 0.80 [95% CI 0.51‐1.26], OS: 96.3% and 91.1%, HR 0.41 [95% CI 0.19‐0.89]).

**Conclusions:**

Short‐ and long‐term outcomes tend to be better in Group A than Group B, indicating that preservation of LCA could be an alternative treatment.

## Graphical abstract

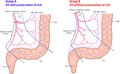

## 1 INTRODUCTION

The Japanese Society for Cancer of the Colon and Rectum (JSCCR) guidelines 2019 for the treatment of colorectal cancer recommend D3 lymph node dissection for clinical stage II/III colorectal cancer.^1^ Still now, during curative resection of sigmoid colon and rectosigmoid colon cancer, it is unclear whether D3 lymph node dissection with the left colic artery (LCA) preservation is beneficial compared to D3 without LCA preservation in terms of clinical outcomes. Choosing whether D3 lymph node dissection with or without LCA preservation depends on a surgeon's preference. LCA preservation is expected to maintain adequate blood supply, which prevents anastomotic leakage. There is a need to determine whether LCA preservation improves clinical short‐ and long‐term outcomes.

JCOG0404 was a randomized controlled trial (RCT) conducted by the Colorectal Cancer Study Group of the Japan Clinical Oncology Group (JCOG) to confirm the non‐inferiority of laparoscopic surgery (LAP) compared to open surgery (OP) for patients with stage II/III colon cancer in terms of overall survival (OS). The surgical treatment of these two groups with or without LCA preservation in the present study required D3 dissection equivalent to complete mesocolic excision with central vascular ligation.^2^ JCOG0404 enrolled more than 1000 patients, making it one of the largest RCTs for patients with colon cancer requiring D3 dissection in Japan. Although non‐inferiority of LAP with D3 dissection to OP for OS could not be confirmed in terms of OS, OS in both groups was similar and better than expected, and laparoscopic D3 surgery could be an acceptable treatment option for patients with stage II or III colon cancer.

At present, there are few reports about the clinical impact of D3 with LCA preservation. We aimed to investigate the clinical impact of D3 lymph node dissection with LCA preservation compared to D3 without LCA preservation by exploratory analyses using the data from JCOG0404. To the best of our knowledge, this is the first study to evaluate the clinical effects of D3 lymph node dissection with or without LCA preservation from collected data of Japanese large‐scale RCT.

## 2 MATERIALS AND METHODS

### 2.1 Summary of JCOG0404

The eligibility criteria of JCOG0404 included histologically proven colon cancer that comprised of adenocarcinoma, signet ring cell carcinoma, or adenosquamous carcinoma; tumor location in the cecum or ascending, sigmoid, or rectosigmoid colon; lesion of T3 or deeper without involving other organs, N0‐2 and M0; ≤8 cm tumor size; and patient age of 20‐75 years. Only accredited surgeons were permitted to perform surgery either as an operator or as an instructor; for OP, surgeons needed to have experience of 30 or more OP colectomies and, for LAP, surgeons needed to have experience of 30 or more cases each of OP and LAP colectomies; and surgeons performing LAP had to be certified according to the Endoscopic Surgery Skill System by the Japan Society for Endoscopic Surgery. D3 lymph node dissection as described below was required. The trial's primary endpoint was OS. In patients with stage II/III colon cancer, non‐inferiority of LAP with D3 dissection to OP for OS could not be confirmed.

JCOG0404 was registered with the UMIN Clinical Trials Registry, number C000000105, and http://ClinicalTrials.gov, number NCT00147134. The details of the JCOG0404 study have been reported elsewhere.^3^, ^4^

The data of patients who received assigned sigmoidectomy and anterior resection in JCOG0404 were used in this exploratory analysis.

### 2.2 Operative methods of D3 dissection with or without LCA preservation

**Figure 1:**
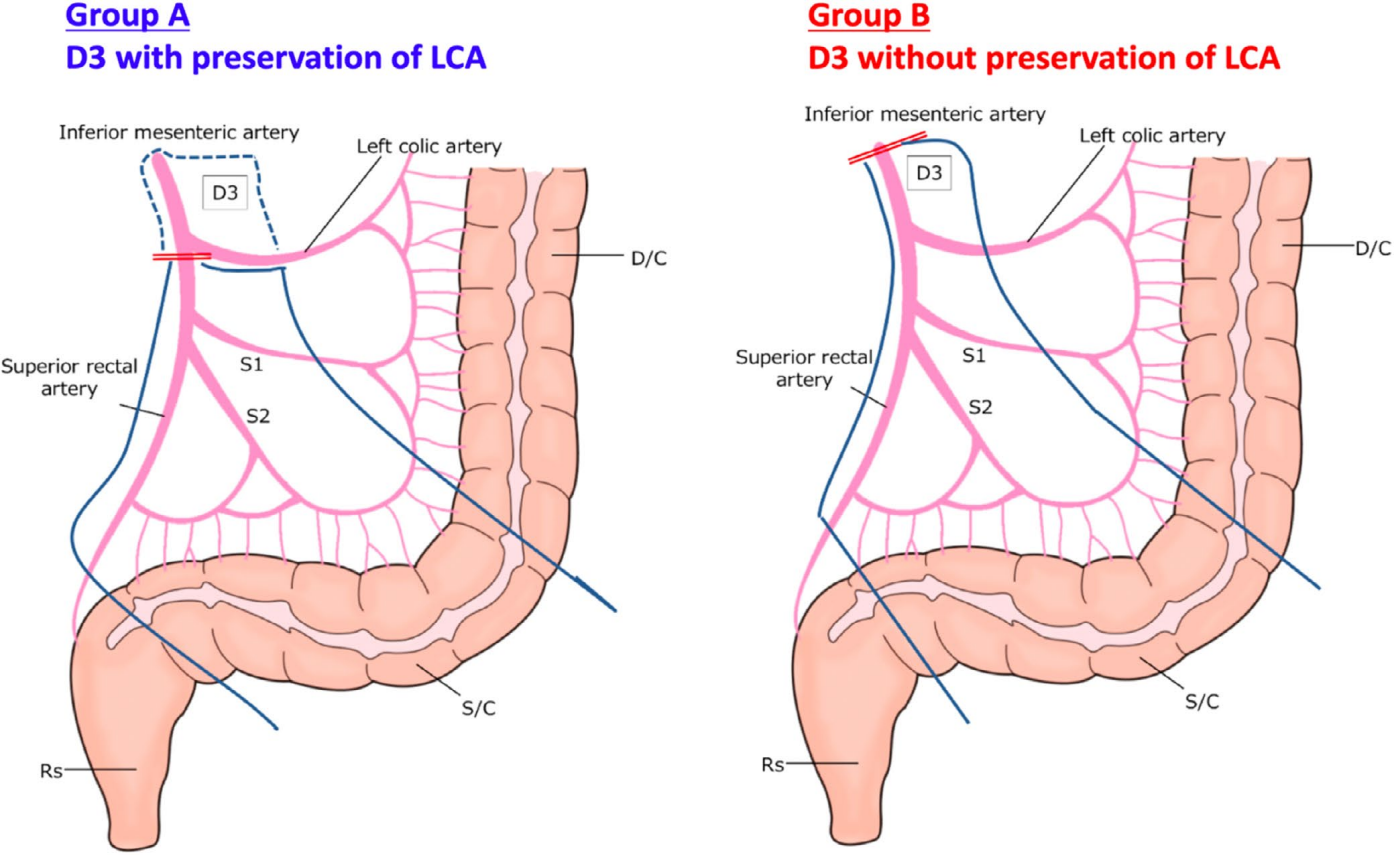
Schema of D3 lymphadenectomy with (Group A) and wihtout (Group B) presearvation of the left coloc artery (LCA)

For left‐sided tumors, removal of lymph nodes at the root of the inferior mesenteric artery was performed along with high ligation (Group B) or with LCA preservation and ligation of the inferior mesenteric artery just distal to the LCA (Group A) (Figure 1). The decision to perform procedures with or without LCA preservation depended on the physician's choice. Procedure type was identified from photographs of the surgical field collected for central surgical review in JCOG0404. Completion of high‐quality surgery with D3 dissection was confirmed in JCOG0404 by central peer review of photographs of the surgical procedures in addition to operator regulations.^5^

### 2.3 Endpoints and statistical considerations

Adverse events were evaluated according to CTCAE 3.0. Postoperative mortality and morbidity were respectively defined as death from any cause and any grade 1 or higher adverse event including anastomotic leakage, paralytic ileus, bowel obstruction, and wound complication within 30 days after surgery. The background characteristics of the patients underwent D3 with LCA preservation were compared with those without LCA preservation. Wilcoxon rank‐sum test for continuous variables and Fisher's exact test for categorical variables were performed to compare the two procedures. We used the Cox proportional hazard model to estimate the hazard ratio (HR) for overall survival (OS) and relapse‐free survival (RFS) of OS and RFS and the Kaplan‐Meier method to estimate OS and RFS. Multivariable Cox regression analysis was conducted for OS and RFS to adjust confounding factors. AS for complications, mutivariable logistic regression analysis was conducted to estimate odds ratio (OR) and its 95% confidence interval (CI). A two‐sided *P* value of <.05 was considered statistically significant. All statistical analyses were performed using SAS ver. 9.4.

[Correction added on 30 March 2020, after the first online publication: “for OS and RFS” has been included in the above paragraph.]

## 3 RESULTS

**Figure 2:**
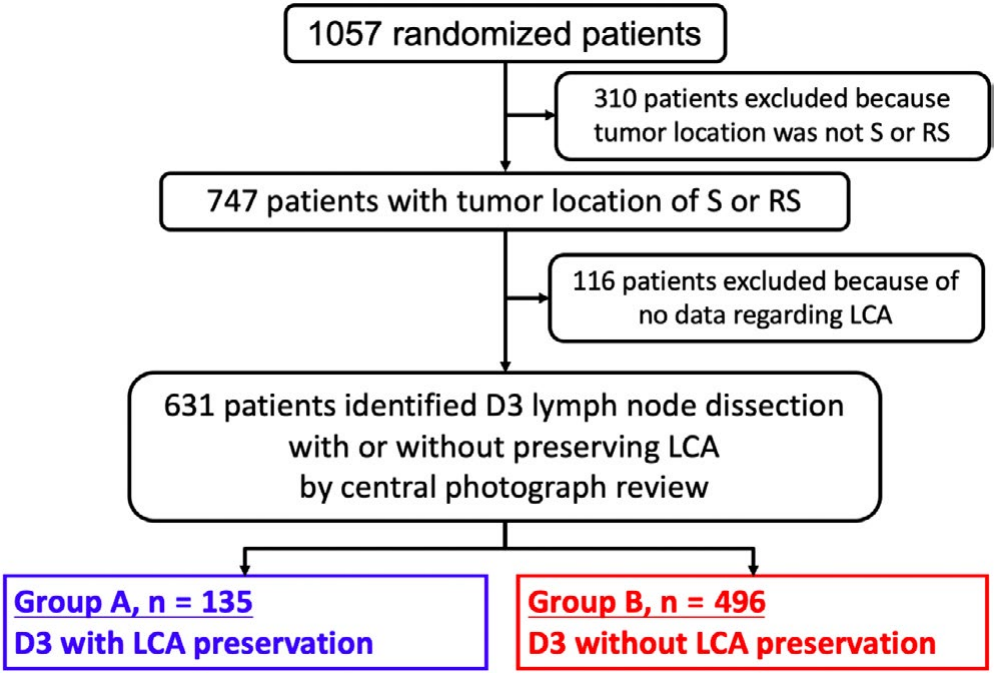
Patient flow diagram

Figure 2 shows the patient flow diagram of the present study. Among all of the 1057 randomized patients, 310 were excluded because tumor location was not in the sigmoid or rectosigmoid colon, leaving 747 patients with a tumor located in the sigmoid or rectosigmoid colon. D3 lymph node dissection with or without LCA preservation was identified according to the photographs of the surgical field collected for central surgical review in JCOG0404. Among the 747 patients, 116 patients were excluded because of lack of data regarding the LCA. Finally, 135 patients underwent D3 with LCA preservation and 496 patients underwent D3 without LCA preservation, and the results were compared and analyzed. With the exception of patient age, there were no significant differences in sex, clinical stage, tumor location, or proportion of laparoscopic surgery between the two groups (Table 1).

**Table 1:** Patient characteristics

	Group A	Group B	Two‐sided *P*
	(n = 135)	(n = 496)	
Sex			
Male	74 (54.8%)	305 (61.5%)	.17
Female	61 (45.2%)	191 (38.5%)	
Age (y) (median, range)	60, 39‐75	64, 28‐75	.05
Clinical stage			
II	98 (72.6%)	338 (68.2%)	.35
III	37 (27.4%)	158 (31.8%)	
Tumor location			
S	86 (63.7%)	325 (65.5%)	.69
RS	49 (36.3%)	171 (34.5%)	
Laparoscopic surgery	64 (47.4%)	247 (49.8%)	.63

Table 2 shows the operative findings. There were no significant differences in operation time or blood loss between the two groups. However, the number of harvested nodes in Group B was significantly higher than in Group A.

**Table 2:** Operative findings

	Group A	Group B	Two‐sided *P*
	(n = 135)	(n = 496)	
Operation time (min)			
Median	185	186	.33
IQR	150‐255	150‐226.5	
Range	72‐465	80‐616	
Blood loss (mL)			
Median	60	50	.53
IQR	20‐130	17.5‐130	
Range	0‐1247	0‐3395	
Number of harvested nodes			
Median	19	21	.01
IQR	14‐24	15‐28	
Range	2‐64	2‐78	

Abbreviation: IQR, interquartile range.

Table 3 shows the operative morbidity and the number of postoperative hospital days of all patients in both groups. Overall postoperative complications of all grades occurred in 13 patients (9.6%) in Group A and 107 patients (21.6%) in Group B, which was significantly different (*P* = .02). Although there were no significant differences in anastomotic leakage, paralytic ileus, or bowel obstruction between the two groups, wound complications in Group B were significantly higher than those in Group A (*P* = .01). The numbers of postoperative hospital days were not different between the two groups. Table 4 showed the multivariate analysis of clinicopathological factors for postoperative Grade 1–4 complications. The OR for Group A was 0.375 (95% CI, 0.201‐0.697, *P* = .0019) by multivariable analysis including approach (open surgery vs laparoscopic surgery), age, sex, body mass index, primary tumor location and stage as covariates.

**Table 3:** Operative morbidity

CTCAE v3.0	Group A (n = 135)		Group B (n = 496)		Two‐sided *P*
	No. of Patients	%	No. of Patients	%	
Postoperative Grade 1 or more complications	13	9.6	107	21.6	.001
Anastomotic leakage	4	3	25	5	.36
Paralytic ileus	3	2.2	19	3.8	.60
Bowel obstruction	0	0	6	1.2	.35
Wound complication	1	0.7	29	5.8	.01
Postoperative hospital days, median	11		11		.82
(IQR) [Range]	(9‐12) [7‐56]		(9‐14) [5‐67]		

Abbreviation: IQR, interquartile range.

**Table 4:** Multivariable analysis of clinicopathological factors for postoperative Grade 1 or more complications

	Postoperative complications		
	Odds ratio	95% CI	Two‐sided *P*
Age			
65≤	1.223	0.809‐1.850	.3396
<65			
Sex			
M/	0.978	0.628‐1.525	.9225
F			
Location			
S	0.502	0.330‐0.763	.0013
RS			
Approach			
Laproscopic	0.629	0.415‐0.953	.0288
Open			
LCA preservation			
With preservation	0.375	0.201‐0.697	.0019
Without preservation			
BMI			
20‐25	1.432	0.715‐2.867	.3111
20≤	1.287	0.692‐2.391	.4254
<25			
cStage III			
cStageIII	1.039	0.670‐1.611	.8644
cStageII			

**Figure 3:**
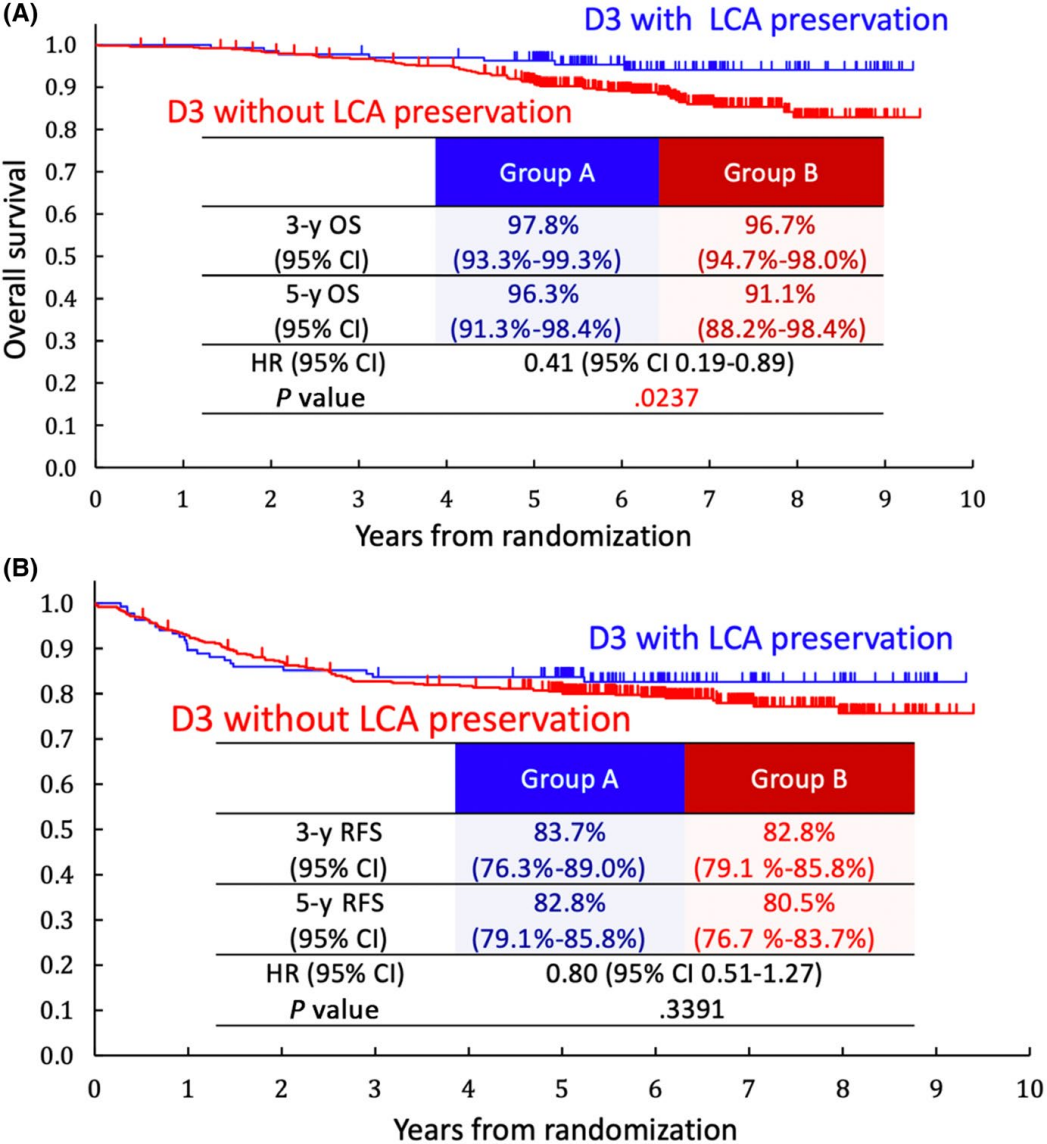
A, Overall survival (OS) rate. B, Relapse‐free survival (RFS) rate. CI, confidence interval; LCA, left colic artery

The estimated 5‐year OS of Group A was 96.3% (95% CI, 91.3%‐98.4%), whereas that of Group B was 91.1% (95% CI, 88.2%‐98.4%). The HR for Group A was 0.41 (95% CI, 0.19‐0.89, *P* = .024) (Figure 3A). The HR for Group A was 0.41 (95% CI, 0.19‐0.90) by multivariable analysis including approach (open surgery vs laparoscopic surgery), age, sex, body mass index, primary tumor location and stage as covariates (Table 5). The estimated 5‐year RFS of Group A was 83.7% (95% CI, 76.3%‐89.0%), whereas that of Group B was 80.5% (95% CI, 76.7%‐83.7%). The HR for Group A was 0.80 (95% CI, 0.51‐1.27, *P* = .34) (Figure 3B). The HR for Group A was 0.83 (95% CI, 0.53‐1.31) by multivariable analysis (Table 5).

[Correction added on 30 March 2020, after the first online publication: “OR” has been amended to “Odds ratio” in the column heading, and a new entry has been added under the sub‐heading BMI]

**Table 5:** Multivariable analysis of clinicopathological factors for overall survival and Relapse‐free survival

	Overall survival			Relapse‐free survival		
	HR	95% CI	*P* value	HR	95% CI	*P* value
Age						
65<	1.130	0.697‐1.832	.6209	0.996	0.699‐1.419	.9819
<65						
Sex						
M/	1.373	0.806‐2.341	.2436	1.490	1.006‐2.205	.0465
F						
Location						
S	0.971	0.584‐1.614	.9102	0.960	0.665‐1.386	.8266
RS						
Approach						
Laproscopic	0.958	0.593‐1.546	.8600	1.125	0.793‐1.596	.5092
Open						
LCA preservation						
With preservation	0.410	0.529‐1.308	.0263	0.830	0.529‐1.308	.4267
Without preservation						
BMI						
25<	0.829	0.415‐1.654	.5944	0.865	0.520‐1.440	.5771
20‐25	1.099	0.515‐2.345	.8062	1.020	0.580‐1.795	.9446
<25						
cStage III						
cStage III	1.187	0.716‐0.5059	.5059	1.340	0.933‐1.924	.1134
cStage II						

[Correction added on 30 March 2020, after the first online publication: The P value of Laproscopic Open is changed from “.860” to “.8600”, and a new entry has been added under the sub‐heading BMI]

## 4 DISCUSSION

Our analysis revealed that the median operative time, median blood loss, and proportion of Grade 1 or higher anastomotic leakage and intestinal paralysis were not remarkably different between the two groups. However, significantly more overall postoperative complications occurred in Group B than in Group A. In terms of efficacy, the 5‐year proportions of OS were better in Group A than Group B. We considered that D3 lymph node dissection with LCA preservation could be an alternative treatment to D3 without LCA preservation.

Generally, there are advantages and disadvantages to both procedures. The advantage in D3 without LCA preservation is en bloc lymph node dissection of the root of the inferior mesenteric artery, which is considered suitable from the viewpoint of preventing the spillage of micrometastatic cells. Its disadvantages include a higher possibility of leakage due to severing of the LCA rather than preserving it and sacrificing of the autonomic nerves around the LCA. In D3 with LCA preservation, on the other hand, the advantage is maintenance of the blood supply, which helps to prevent anastomotic leakage and intestinal paralysis. Its disadvantages include the possibility of spillage of micrometastatic cells because of skeletonization of the LCA and the requirement of a more complicated procedure with longer operation time than that without LCA preservation.^6^

In previous reports of retrospective studies of sigmoid colon and rectal cancer that compared D3 with LCA preservation to D3 without LCA preservation, Yasuda et al reported that there were no significant differences in terms of short‐ and long‐term outcomes between the two procedures.^7^ In addition, Sekimoto et al showed that there were no differences in terms of short‐term outcomes including operation time, blood loss, and number of harvested lymph nodes between the two procedures.^8^ However, several reports showed that precise staging was performed by D3 without preservation of the LCA.^9^, ^10^ Even now, there is still no consensus on the level of arterial ligation in sigmoid and rectosigmoid colon cancer. To the best of our knowledge, the present study is first to show the clinical outcomes of D3 with LCA preservation compared to D3 without LCA preservation from large‐scale data collected in a multi‐institutional RCT. The present study demonstrated LCA preservation might be a beneficial factor for better short‐term outcomes.

The present study revealed that, in terms of postoperative complications, short‐term outcomes were better in patients undergoing D3 with LCA preservation than D3 without LCA preservation. Although it is generally considered that D3 with LCA preservation is a more complicated technique requiring longer operation time than D3 without LCA preservation, the present study found no significant differences in terms of operation time and blood loss. This is why the present study did not need to take into consideration the surgical learning curve as a potential risk factor because of the study chair of JCOG0404 certified surgeons at each participating institution according to the aforementioned criteria. In terms of the lower incidence of overall complications and wound complications in Group A, we speculated that preserving the LCA enabled preservation of the autonomic nerves, drainage vein, and the immune system as well as the LCA, which might be associated with lower incidence of anastomotic leakage, paralytic ileus, bowel obstruction, and wound complication.

In terms of OS, the present study showed that D3 with LCA preservation was significantly better than D3 without LCA preservation. As previous studies reported, fewer postoperative complications might contribute to a better prognosis.^11^, ^12^ Although the number of harvested lymph nodes in D3 with LCA preservation was lower than that in D3 without LCA preservation, we considered it sufficient to dissect D3 lymph nodes oncologically through D3 with LCA preservation because the present study revealed the non‐inferiority of D3 with LCA preservation in terms of prognosis compared with D3 dissection without LCA preservation. Although it is unknown whether the D3 lymph node dissection is necessary or not, JSCCR guideline recommends the D3 lymph node dissection. In the present study, we evaluated which was a better procedure for D3 dissection, with or without LCA preservation. The HR for Group A of 5‐year OS was 0.41, which might mean that preservation of LCA strongly contributed to better survival.

There are a few limitations in this study. First, this study is an exploratory subgroup analysis of data from a RCT. Thus, the decision to perform procedures with or without preservation of the LCA depended on physician preference, which were almost decided by institutional policy and might affect clinical outcomes. Second, the number of pathological T3 or less (ss or shallower) tumors in Group A was significantly higher than that in Group B (88.9% vs 80.4%, *P* = .023, data was not shown), although there were no significant differences in terms of pathological N status between the two groups. Therefore, comparability might not be maintained even though multivariable analysis showed similar HR compared with univariable analysis. Thus, further investigation is necessary to precisely evaluate the usefulness of D3 lymph node dissection with LCA preservation.

In conclusion, our analysis for stage II/III sigmoid and rectosigmoid colon cancer found that short‐ and long‐term outcomes were better in D3 dissection with preservation of the LCA than D3 dissection without preservation of the LCA. We concluded that D3 lymph node dissection with preservation of the LCA could be an alternative treatment for D3 lymph node dissection.

## DISCLOSURE

Conflict of Interest: All authors have no conflicts of interest to declare.

## References

[1] Hashiguchi Y , Muro K , Saito Y , Ito Y , Ajioka Y , Hamaguchi T , et al. Japanese Society for Cancer of the Colon and Rectum (JSCCR) guidelines 2019 for the treatment of colorectal cancer. Int J Clin Oncol. 2020;25(1):1–42. 31203527 10.1007/s10147-019-01485-z PMC6946738

[2] Kitano S , Inomata M , Sato A , Yoshimura K , Moriya Y , Japan Clinical Oncology Group Study . Randomized controlled trial to evaluate laparoscopic surgery for colorectal cancer: Japan Clinical Oncology Group Study JCOG 0404. Jpn J Clin Oncol. 2005;35(8):475–7. 16006574 10.1093/jjco/hyi124

[3] Kitano S , Inomata M , Mizusawa J , Katayama H , Watanabe M , Yamamoto S , et al. Survival outcomes following laparoscopic versus open D3 dissection for stage II or III colon cancer (JCOG0404): a phase 3, randomised controlled trial. Lancet Gastroenterol Hepatol. 2017;2(4):261–8. 28404155 10.1016/S2468-1253(16)30207-2

[4] Yamamoto S , Inomata M , Katayama H , Mizusawa J , Etoh T , Konishi F , et al. Short‐term surgical outcomes from a randomized controlled trial to evaluate laparoscopic and open D3 dissection for stage II/III colon cancer: Japan Clinical Oncology Group Study JCOG 0404. Ann Surg. 2014;260(1):23–30. 24509190 10.1097/SLA.0000000000000499

[5] Hino T , Okajima M , Ikeda S , Yoshimitsu M , Ohdan H , Watanabe M . Effect of left colonic artery preservation on anastomotic leakage in laparoscopic anterior resection for middle and low rectal cancer. 2008 Abstract book of 2008 ELSA (Endoscopic and Laparoscopic. Surgeons of Asia) September 5–6, Yokohama Japan Abstract number ES27‐3, p 33.

[6] Nakajima K , Inomata M , Akagi T , Etoh T , Sugihara K , Watanabe M , et al. Quality control by photo documentation for evaluation of laparoscopic and open colectomy with D3 resection for stage II/III colorectal cancer: Japan Clinical Oncology Group Study JCOG0404. Jpn J Clin Oncol. 2014;44(9):799–806. 25084776 10.1093/jjco/hyu083

[7] Yasuda K , Kawai K , Ishihara S , Murono K , Otani K , Nishikawa T , et al. Level of arterial ligation in sigmoid colon and rectal cancer surgery. World J Surg Oncol. 2016;14:99. 27036117 10.1186/s12957-016-0819-3 PMC4818479

[8] Sekimoto M , Takemasa I , Mizushima T , Ikeda M , Yamamoto H , Doki Y , et al. Laparoscopic lymph node dissection around the inferior mesenteric artery with preservation of the left colic artery. Surg Endosc. 2011;25(3):861–6. 20725744 10.1007/s00464-010-1284-7

[9] Titu LV , Tweedle E , Rooney PS . High tie of the inferior mesenteric artery in curative surgery for left colonic and rectal cancers: a systematic review. Dig Surg. 2008;25(2):148–57. 18446037 10.1159/000128172

[10] Kanemitsu Y , Hirai T , Komori K , Kato T . Survival benefit of high ligation of the inferior mesenteric artery in sigmoid colon or rectal cancer surgery. Br J Surg. 2006;93(5):609–15. 16607682 10.1002/bjs.5327

[11] Aoyama T , Oba K , Honda M , et al. Impact of postoperative complications on the colorectal cancer survival and recurrence: analyses of pooled individual patients' data from three large phase III randomized trials. Cancer Med. 2017;6(7):1573–80. 28639738 10.1002/cam4.1126 PMC5504309

[12] Shimada H , Fukagawa T , Haga Y , Oba K . Does postoperative morbidity worsen the oncological outcome after radical surgery for gastrointestinal cancers? A systematic review of the literature. Ann Gastroenterol Surg. 2017;1(1):11–23. 29863169 10.1002/ags3.12002 PMC5881350

